# Media Forensics Considerations on DeepFake Detection with Hand-Crafted Features

**DOI:** 10.3390/jimaging7070108

**Published:** 2021-07-01

**Authors:** Dennis Siegel, Christian Kraetzer, Stefan Seidlitz, Jana Dittmann

**Affiliations:** Department of Computer Science, Otto-von-Guericke University, 39106 Magdeburg, Germany; stefan.seidlitz@ovgu.de (S.S.); jana.dittmann@ovgu.de (J.D.)

**Keywords:** DeepFake detection, hand-crafted features, forensic process model, plausibility of decisions

## Abstract

DeepFake detection is a novel task for media forensics and is currently receiving a lot of research attention due to the threat these targeted video manipulations propose to the trust placed in video footage. The current trend in DeepFake detection is the application of neural networks to learn feature spaces that allow them to be distinguished from unmanipulated videos. In this paper, we discuss, with features hand-crafted by domain experts, an alternative to this trend. The main advantage that hand-crafted features have over learned features is their interpretability and the consequences this might have for plausibility validation for decisions made. Here, we discuss three sets of hand-crafted features and three different fusion strategies to implement DeepFake detection. Our tests on three pre-existing reference databases show detection performances that are under comparable test conditions (peak AUC > 0.95) to those of state-of-the-art methods using learned features. Furthermore, our approach shows a similar, if not better, generalization behavior than neural network-based methods in tests performed with different training and test sets. In addition to these pattern recognition considerations, first steps of a projection onto a data-centric examination approach for forensics process modeling are taken to increase the maturity of the present investigation.

## 1. Introduction

DeepFakes (a neologism combining the terms “deep learning” and “fake”) are synthetic videos (or images) in which a person’s face (and optionally also voice) is replaced with someone else’s likeness using deep learning technologies. Having emerged in late 2017, DeepFakes nowadays pose a serious threat to the trust placed in video footage. Papers such as [1,2] elaborate on the effect of DeepFakes on current politics, disinformation and trust.

Like countering any other form of image, audio or video manipulation, detecting DeepFakes is an important task for media forensics and is currently receiving a lot of research attention due to the significance of the threat.

According to a well established definition given in [3], information technology (IT) forensics is: “*The use of scientifically derived and proven methods toward the preservation, collection, validation, identification, analysis, interpretation, documentation and presentation of digital evidence derived from digital sources for the purpose of facilitating or furthering the reconstruction of events found to be criminal,* […]”.

This paper focuses on DeepFake detection as a novel challenge in the IT forensics subdiscipline of media forensics. In contrast to many other forensic subdisciplines, such as, e.g., the field of fingerprint analysis, this field is an especially young and immature research field, currently being far away from achieving the ultimate goal of courtroom readiness.

Regarding the basic methodology applied in the state-of-the-art work in DeepFake detection, it can be stated that most of the current research work is based on pattern recognition approaches using feature spaces learned with the help of neural networks. While this method achieves promising detection rates for small scale empirical evaluations with selected DeepFake datasets, it has the inherent drawback that it is extremely hard to validate the plausibility of decisions made by a neuronal network since the semantics of the features learned cannot easily be interpreted by humans. For other, more established, pattern recognition disciplines such as template matching or statistical pattern recognition, the issue of plausibility testing also exists, because the results generated by the application of machine learning strategies lack the intuitive verification that usually accompanies human decision-making processes. Nevertheless, for these disciplines, validation methods have been developed over the decades to establish whether the results of the learning and decision processes are reasonable. In practice, this means to establish that the patterns trained and detected are really the patterns that the user wants to distinguish between and that side-effects as well as external influence factors are known for the pattern recognition process. Such methods, which include, amongst others, feature selection strategies, as well as model analysis methods aimed at establishing the exact decision (or detection) performance and error behavior of an analysis method. The reason to do this is that this knowledge determines the plausibility of the result of the application of pattern recognition mechanisms in a forensic application scenario and should therefore be directly linked to the trust we place in their decisions.

In addition to the problems in estimating the plausibility of decisions of current (mostly neural network-driven) DeepFake detection methods, a second shortcoming in the current state of the art in this field has to be mentioned here: Apart from the considerations of efficiency (i.e., detection performance and plausibility), all forensic methods should aim at fulfilling some form of forensic conformity. Criteria for such conformity should address the admissibility of methods as a basis for expert witnesses’ testimony as evidence in legal proceedings. For the United States of America (by far the most active legal system worldwide), those criteria are codified, amongst other regulations, by the so called Daubert standard (see e.g., [4] or [5] for a detailed discussion of this US case-law standard) in combination with the US Federal Rules of Evidence (FRE) [6]. In addition to those admission criteria for expert witnesses’ testimony questions of evidence handling (i.e., chain of custody) also have to be looked into.

To address aspects of these two identified shortcomings (i.e., the explainability issues of feature spaces learned using a neural network on one hand and the lack of adherence to forensic process models on the other hand), this paper provides the following two main contributions:

- Using hand-crafted features for DeepFake detection and comparison with the performance of state-of-the-art deep learning-driven approaches, we discuss three sets of hand-crafted features and three different fusion strategies to implement DeepFake detection. Those features analyze the blinking behavior, the texture of the mouth region as well as the degree of texture found in the image foreground. Our tests on three pre-existing reference databases show detection performances that are under comparable test conditions to those of state-of-the-art methods using learned features (in our case obtaining a maximum AUC of 0.960 in comparison to a maximum AUC of 0.998 for a recent approach using convolutional neural networks). Furthermore, our approach shows a similar, if not better, generalization behavior (i.e., AUC drops from values larger than 0.9 to smaller than 0.7) than neural network based methods in tests performed with different training and test sets.
  In addition to those detection performance issues, we discuss at length that the main advantage which hand-crafted features have over learned features is their interpretability and the consequences this might have for plausibility validation for decisions made.
- Projection onto a forensic process model: With the aim to improve the maturity of pattern recognition-driven media forensics, we perform first steps of the projection of our work onto an established forensic process model. For this, a derivative of the forensic process model for IT forensics published in 2011 by the German Federal Office for Information Security (BSI) is used here. This derivative, or more precisely extension, is called the Data-Centric Examination Approach (DCEA) and has seen its latest major overhaul in 2020 in [7]. While it is not yet perfectly capable of fitting the needs of media forensics analyses, our work shows first benefits of this modeling as well as points where DCEA would need to undergo further extension to fit those purposes.

The paper is structured as follows: In Section 2, the background and state of the art in DeepFake detection (Section 2.1), feature space design alternatives (Section 2.2) and the forensic process model chosen for this paper (Section 2.3) are discussed. Section 3 discusses the chosen solution concept for implementing DeepFake detection with hand-crafted features, while Section 4 focuses on implementation details.

Section 5 presents and discusses our evaluation results, structured into results for individual detectors (Section 5.1) and for fusion operators (Section 5.2). In Section 6, we provide a summary of the results and a comparison with other approaches from the state of the art (in Section 6.1) as well as our conclusion on the comparison between hand-crafted and learned features for DeepFake detection (in Section 6.2). Section 7 closes the paper with some indication for potential future work.

## 2. Background and State of the Art

By arguing that "Multimedia Forensics is not Computer Forensics", the authors of [8] point out that “*multimedia forensics and computer forensics belong to the class of digital forensics, but they differ notably in the underlying observer model that defines the forensic investigator’s view on (parts of) reality,* […] *while perfect concealment of traces is possible for computer forensics, this level of certainty cannot be expected for manipulations of sensor data*”. Even though this statement dates back to 2009, before the rise of neural network-driven data generation methods, such as generative adversarial networks (GANs), it still holds true; additionally, modern-day targeted media manipulations such as DeepFake generation, either leave telltale traces of the manipulation (here, the synthesis and insertion of a face into a video) or violate the source characteristics (e.g., violating the noise pattern of the camera). Recent papers on DeepFake detection, such as [9], provide strong indication that, if applied correctly, targeted detection using pattern recognition methods might be a viable media forensics approach to counter DeepFakes.

In Section 2.1 of this chapter, the state of the art regarding recent DeepFake detection methods is briefly summarized. Following this survey, which points out that nearly all recent methods found in the literature are looking at learned feature spaces as a means of tackling this pattern recognition problem, Section 2.2 discusses the existing alternatives for feature space design and reflects upon their suitability in sensitive decision processes, such as e.g., medical image processing or (media) forensics. Additionally, in Section 2.3, a discussion on the needs for integration of pattern recognition-driven methods into a forensic process model is summarized.

### 2.1. DeepFake Detection

Usually, the detection of DeepFakes happens with various combined Convolutional Neural Network (CNN) architectures such as autoencoders (AEs). The reasons behind this are obvious: First, most DeepFakes are produced with AEs because internet platforms such as YouTube provide many video sources with different human faces which are usable for the training of DeepFake generators based on neural networks. FakeApp [10] is one example of an autoencoder–decoder structure which is able to swap the latent features of two different faces [11]. These architectures introduce several artifacts to the video while creating a DeepFake that are, in most cases, not visible for the human eye but are potential artifacts that could be utilized for DeepFake detection using image or video analysis methods. It stands to reason that neural networks are also useful for the detection of DeepFake videos, assuming that there is a sufficiently large set of representative data to train features, allowing for the localization of the aforementioned artifacts. Second, which is also a consequence of the first reason, several large and publicly available DeepFake databases (such as FaceForensic++ [12] or Celeb-DF [13]) already exist and provide huge datasets, which can easily be used for the training of CNN-based DeepFake detectors.

The survey paper from Nguyen et al. [11] summarizes different DeepFake detection approaches into the two main categories of *temporal features across video streams* (i.e., inter-frame analysis) and *visual artifacts within video frames* (i.e., intra-frame analysis). For example, the approach of Sabir et al. [14] extracts temporal features of video streams for the detection of DeepFake videos: The authors analyze a potential DeepFake video frame-by-frame for low level artifacts which are only present in single frames to class a video as a DeepFake. Then, they use a Recurrent Convolutional Network (RCN) model to detect and track the temporal artifacts across frames [11,14]. In Li et al.’s work [15], another CNN-based inter-frame analysis approach addresses the eye blinking of a person in a video under the assumption that many DeepFake generated videos are not able to reproduce a natural blinking behavior. The authors first extract the eye areas based on six eye landmarks from a segmented face region. After that, they use the extracted eye area of all video frames in a long-term recurrent convolutional network (LRCN) to detect temporal discrepancies in the blinking behavior [11,15]. An approach which should also be considered for these temporal features across video streams category is described in [16]. Here, the authors analyze (amongst other detection strategies) the lip movements with a combined neural network structure of Mel-Frequency Cepstral Coefficients (MFCCs), Principal Coefficients (PCAs) and an RNN-based (recurrent neural network) Long Short-Term Memory (LSTM) and check whether the lip movement is synchronized to the audio signal [16,17].

The second category for DeepFake detectors, defined by Nguyen et al. [11] (i.e., the intra-frame analyses), is divided into the subcategories of deep and shallow classifiers: During the DeepFake creation process, it is necessary to warp the face area by scaling, rotation and shearing. Deep classifiers address resolution inconsistencies between the warped face area and the surrounding context. These inconsistencies are represented in artifacts which are detectable by CNNs (see, e.g., [11,18]). In contrast, the so called shallow classifiers refer to different visual feature artifacts in head pose, eyes, teeth or in facial contours. In particular, the last three features are addressed in Matern et al.’s work [19]. They solve the DeepFake detection by analyzing the eye and teeth areas for missing reflections or details as well as the texture features from the facial region [11,19].

Other survey papers in this rapidly growing research field, such as the work of, e.g., Yu et al. [20], use the main structure of the DeepFake detection method to classify these methods into several detector categories. Similar to Nguyen et al., they distinguish broadly between inter- and intra-frame analyses. In their scheme, the first (i.e., temporal) features are covered by temporal consistency-based methods using mainly network structures such as recurrent CNNs which are able to detect temporal features frame-by-frame. The latter category is addressed by general network-based methods, which are divided into transfer learning methods and specially designed networks. The methods of transfer learning re-train detectors originally trained for a different recognition problem, while specially designed networks construct and train entirely novel architectures and detectors dedicated entirely to the task of detecting DeepFake videos.

In summary of the (survey) papers discussed above, it can be stated that most DeepFake detection approaches are based on (convolutional) neural networks to learn the feature space to be used. This approach usually requires big databases of real and DeepFake videos to generate detectors that usually perform with very high detection rates on test material that is similar to the used training material in terms of its characteristics.

Hand-crafted feature methods, as an alternative to features learned with neural networks, have the benefit that they (at least theoretically) could work without training. In addition to this and other potential benefits (see Section 2.2), hand-crafted feature spaces for the detection of DeepFake videos are much less common in the literature than neural network-based approaches. Most of the existing research papers relying on hand-crafted approaches (such as [21,22,23]) use Support Vector Machines (SVMs) for a fast and efficient detection of DeepFake videos.

For the DeepFake detection of persons of interest (POIs) such a Barack Obama, Hillary Clinton or Donald Trump, Gu et al. [23] analyzed speech in combination with face and head movements. They followed the assumption that a person has individual facial expressions and head movements while they are speaking. Their detection pipeline starts with a single video were they tracked facial and head movements first. These facial expressions are defined by 2D and 3D facial landmark positions and several facial action units which are then used for further evaluation steps. For the DeepFake detection, they trained and tested one-class SVMs only with extracted features from authentic videos of specific POIs.

Jung et al. [24] present a hand-crafted DeepFake detector called DeepVision [24], which evaluates eye blinking behavior. In their first step, they extract the face region from a potential DeepFake video. In the following, they use an eye tracker to detect the eye area of a person. After this step they check the eye area of each frame for closed or open eyes and calculate the eye blink elapsed times and eye blink periods.

Unfortunately, the authors of some survey papers, such as [25,26], refer to learned features using specially designed networks (such as those proposed in [15,18]) and also as being "hand-crafted". This is not our perspective of hand-crafted features because they only design the neural network architectures and not the actual features and their semantics. In the following section, we will provide working definitions for the terms hand-crafted and learned features to be used in this paper.

### 2.2. Feature Space Design Alternatives

In pattern recognition, feature extraction starts from an initial set of input data and builds derived values (features) intended to be informative and non-redundant, facilitating the subsequent learning and generalization steps. It is generally seen to be one form of dimensionality reduction projecting the input into an easier to process and (optimally) less noisy representation. In applied pattern recognition, there generally exist two distinct approaches for feature design:

(a) Features are especially designed (so-called hand-crafted) by domain experts for an application scenario in a process, which, despite the fact that it is sometimes also called intuition-based feature design, usually requires strong domain knowledge. Here, the domain expert uses his/her own experience to construct the features to encode his/her own knowledge about the semantics (and internal as well as external influence factors) inherent to the different pattern classes in the problem at hand. As a result, usually rather low-dimensional feature spaces are designed, which require only small sets of training data (or none at all) for the training (i.e., adaptation/calibration) to a specific application scenario. The semantic characteristics intrinsic to these feature spaces can easily be exploited to validate decisions made using such a feature space.
  Such features can also be the result of the transfer of features from other, related or similar pattern processing problems.
(b) Feature spaces are *g* by methods such as neural networks, where a structure (or architecture) for the feature space is designed (or chosen from a set of known goods) and then labelled training specimens are used to train the network from scratch or re-train an already existing network in transfer learning. The inherent characteristic of this process is that it requires very large sets of labelled, representative data for the training of the network (a little less so in case of transfer learning). The resulting feature spaces and trained models usually lack the encoding of easily interpretable semantics.

While neural network-based methods have seen a growing popularity in the field of media forensics in the last few years, they are still burdened by the problem that the plausibility of a decision made on the basis of such features is extremely hard to verify. One of the main reasons for this is the fact that the learned features as such hardly ever encode semantics that could be interpreted by a human expert. Instead, with the help of decision validation approaches such as the expert interpretation of heatmaps using methods such as Layer-wise Relevance Propagation (LRP; [27]), it can be shown that these methods assign meaning to regions in the input (see e.g. [28]).

For this reason, i.e., problems with the interpretability of the feature space and corresponding decisions, many application fields with sensitive tasks are hesitant to rely on learned features. A good example of a very thorough discussion of the pros and cons of hand-crafted features in contrast to those learned using convolutional neural networks can be found in Lin et al.’s work [29]. In this paper, the authors discuss this issue for specific medical data analysis problems, which, similar to forensics, is another very sensitive research field applying pattern recognition. In their work, they highlight and demonstrate with their datasets three main drawbacks of neural network-based feature space learning:

1. In the case of only small amounts of training data being available (which seems to be a problem encountered often in medical data analysis problems, including clinical studies where “*the recruitment of a large number of patients or collection of large number of images is often impeded by patient privacy, limited number of disease cases, restricted resources, funding constraints or number of participating institutions*” [29]), the classification performance of hand-crafted features (which usually show persistent detection performances with small training datasets) outperformed their feature spaces learned by neural networks. This is hardly astonishing since it is well known that CNNs require a large amount of training data for reliable imaging classification. This situation changes with increasing training dataset sizes.
2. Another advantage of hand-crafted features is interpretability. Lin et al. summarize this issue as follows: “*Therefore, interpretability of* [hand-crafted] *features reveal why liver* [magnetic resonance] *images are classified as suboptimal or adequate*” [29], i.e., these features allow for expert reasoning on errors, loss or uncertainty in decision making.
3. Feature selection strategies help learning about significance and contextual relationship for hand-crafted features, while they fail to produce interpretable results for learned features.

For the more traditional feature space designs (i.e., using hand-crafted features), the question of plausibility verification is usually easier to address. A multitude of methods for feature space analysis have been discussed in the past, including feature space-driven plausibility validation as well as model-driven validation.

Initially, there existed two main approaches for feature selection: wrapper methods, in which the features are selected using the classifier, and filter methods, in which the selection of features is independent of the classifier used. Around 2001, both main approaches were joined into a so-called hybrid method (see, e.g., [30,31]), which are usually used nowadays to analyze hand-crafted feature spaces.

### 2.3. A Data-Centric Examination Approach for Incident Response and Forensic Process Modeling

Forensic process models are an important cornerstone in science and more importantly the practice of forensics. They guide investigations and make them comparable, reproducible as well as certifiable. Usually, the adherence to strict guidelines (i.e., process models) is regulated within any legal system (e.g., in the US by the fourth Daubert criterion (“*the existence and maintenance of standards and controls*” [4])). For mature forensic sciences, such as, for example, dactyloscopy, internationally accepted standards (such as the ACE-V process model for dactyloscopy) have been established over recent decades.

Due to the fact that IT forensics is a rather young discipline in this field (with media forensics being an even younger subdiscipline), it is hardly astonishing that here the forensic process models have not yet achieved the same degree of maturity as in other fields. Nevertheless, they would still be important to achieve universal court acceptability of methods. One well established forensic process model for IT forensics is the one proposed by the German Federal Office for Information Security (BSI). When it was originally published in 2011, its sole focus was on computer and network forensics, but since then it has evolved to also include suite and also some extend the needs of other subdisciplines such as digitized forensics. The latest major revision of this process model, which is used within this paper, can be found in [7] and is called the Data-Centric Examination Approach (DCEA). The core of the DCEA consists of three main aspects: a model of the *phases* of a phase-driven forensic process, a classification scheme for *forensically relevant data types* and *forensic method classes*.

The DCEA phases are briefly summarized in Table 1.

One important reason for this paper to use the DCEA to model our own work is the separation of preparation steps in an investigation into two distinct phases (the strategic preparation (SP) on one hand an the operational preparation (OP) on the other). In [7], the SP is generally defined as: “*The strategic preparation* […] *includes all preparation procedures taken ahead of the actual occurrence of a specific incident*”. Exemplary measures for SP in the context of digital forensics are given by [7] as: “*Documentation and extension of knowledge of IT systems specifics, tool testing for forensic data types and sets of methods determination for error loss and uncertainty estimation, setup of logging capabilities, performance of system landscape analysis, data protection considerations,* […]”. In contrast, the OP is specified to “[…] *include all preparation procedures taken after of the actual occurrence of a specific incident. Those procedures by definition do not alter any data on the targeted system*”. These preparation phases are then followed by the actual application of forensic procedures, separated in DCEA into the triplet of data gathering (DG), data investigation (DI) and data analysis (DA). The whole process is, in every phase (including SP and OP), supported by accompanying documentation, which is in the last phase (documentation (DO)) used as basis for the generation of the official documents regarding the investigation (e.g. the evidence to be interpreted in expert testimony in a court case).

The second core aspect of DCEA is the classification scheme for forensically relevant data types, as summarized in Table 2. The categories in this scheme are not classes in a mathematical sense, since all other later data types are interpreted out of raw data. More recent publications, such as [33], have shown that this scheme needs to be extended accordingly if new investigation domains are considered.

This original set of data types, which was designed with digital (IT) forensics in mind, needs to be adapted to every investigation domain. In [7,32], such an adaptation for the field of digitized forensics has been discussed for the field of dactyloscopy (forensic fingerprint analysis and comparison). This adaptation is summarized in Table 3 below because it is much closer to the requirements we face within this paper than the original data types summarized in Table 2.

The third core aspect of DCEA is the definition of forensic method classes as presented in Table 4. For a detailed discussion on these method classes, including considerations on the estimation of availability in certain investigation contexts, practicalities of the forensic process, etc., we refer to [7].

The DCEA is relevant for the work presented in this paper for two different reasons: On one hand, we will use it in Section 3 to provide a comparative description of the solution concept to address the issue of DeepFake detection in this paper. On the other hand, we will elaborate on the question related to how well this process model fits the needs of media forensics investigations and which changes or extensions would be required in DCEA to provide better support for this very young subdiscipline in IT forensics.

## 3. Solution Concept for DeepFake Detection with Hand-Crafted Features

The main findings considering the background and state of the art in Section 2 can be summarized as follows: DeepFake detection is a very active research field trying to address a significant recent threat. While many detection approaches have been published in the last few years (some reporting astonishing detection performances), only a small number of publications have been tackling the questions of interpretability and plausibility of results. We attribute this lack of studies mainly to the type of features used in the majority of the research published so far, which rely on neural networks to learn feature spaces used, a method that has inherent difficulties with interpretability (see Section 2.2). Additionally, this question of creating the feature spaces required for a pattern recognition-driven media forensics method such as DeepFake detection, a close integration of forensic procedures and “*the existence and maintenance of standards and controls*” [4] is an open issue. This can contribute to the comparative novelty of many media forensics methods, including DeepFake detection.

To address both of these apparent gaps (interpretability of feature spaces and projection into forensic procedures), our work in this paper focuses on the usage of hand-crafted features for this pattern recognition problem as well as discussions on the applicability of the Data-Centric Examination Approach (DCEA, see Section 2.3) to map out our work.

Regarding the pattern recognition aspects, the concept in this paper focuses on four items:

- The design, implementation and empirical evaluation of features for DeepFake detection: Here, two feature spaces hand-crafted especially for DeepFake detection and a hand-crafted feature space derived from a different but similar pattern recognition problem domain (face morph detection) are implemented and evaluated. For the empirical evaluation, pre-existing reference databases containing DeepFake as well as benign ("original") face video sequences are used together with a pre-existing out of the box classification algorithm implementation. To facilitate the interpretation of results and the comparability with other detector performances reported in the state of the art, different well established metrics are used: detection accuracy, Cohen’s kappa as well as (ROC) AUC (Area Under the Curve (of the Receiver Operating Characteristic)).
- The discussion of different information fusion techniques and the empirical comparison with detection performances of individual classifiers: Here, with feature-level fusion and decision-level fusion, two different concepts are applied. For the latter, with the majority voting and weighted linear combination, two popular choices are used and compared with single classifiers in terms of the classification performance achieved.
- The comparison of the detection performance of our hand-crafted features with performances of learned feature spaces from the state of the art in this field: Here, the results obtained by single classifiers as well as fusion approaches are compared in terms of detection accuracy with different approaches from the state of the art, relying on learned features.
- Attempts at validating the detectors’ decisions on basis of the features and trained models: Some classifiers, such as the decision tree algorithm used in this paper, train models that can be read, interpreted and compared by humans. Here, we analyze the decision trees trained on different training sets to identify the most relevant features and see how much these trees have in common and where they differ.

In addition to these pattern recognition aspects, we project the different operational aspects in training, validating and applying the DeepFake detectors into the established process model DCEA to show how such media forensics methods would have to be integrated into forensic procedures. In this projection, the first question to be asked concerns where the detector is supposed to be used. There exist two potential operation points in the phases described by the DCEA: either as a method of explicit means of intrusion detection (EMID) as part of incident detection mechanisms, which would place the whole DeepFake detection with the training of the method and its application into the phase of strategic preparation (SP), or in scaling of methods for evidence gathering (SMG), which would place DeepFake detection after an incident is detected or suspected and place the corresponding components in the operational preparation (OP), data gathering (DG), data investigation (DI) and data analysis (DA) phases. These two distinct operation points as a live detector or as means of post-mortem (or a posterior) analysis in data investigation have, amongst other effects, significant impact on the training scenario that can be assumed: In the case of application as an live detector (EMID), in SP, only pre-trained models can be applied. In the case of a post-mortem (SMG) detector, in the OP the material to be investigated can be analyzed to design targeted training datasets perfectly matching the characteristics encountered. Using those sets (and own DeepFake algorithms to also generate a specimen for this class) optimal models could be trained for each case. In this paper, the conceptual choice made is that of a live detector, reserving considerations on targeted training for future work.

The concept of training brings us to a second issue where the principles of the DCEA can help structuring of the description of media forensics methods such as DeepFake detectors: The accompanying documentation in the DCEA is meant to allow for interpretability and plausibility validation steps while compiling the case documentation in DO. For our work, this implies not only documenting all details of the pattern recognition process at hand but also using these data to reason about the plausibility of decisions (e.g., by comparing the characteristics of training and test sets to determine questions of generalization power).

One important realization when trying to apply the DCEA data types for digital or digitized forensics, as summarized in Table 2 and Table 3, is that they do not perfectly match the media forensics task at hand. Using the original model for digital forensics, only four of the data types would be covered (raw data differentiated into different user data media streams (video, audio, network stream) and possibly hardware data (derived from the camera/microphone used) as well as details about data). If the model for digitized dactyloscopy is used, which slightly better matches with the characteristics of our application scenario, then eight of the ten data types would be directly relevant (processed signal data (DD2), contextual data (DD3), parameter data (DD4), trace characteristic feature data (DD5), model data (DD7), classification result data (DD8), chain of custody data (DD9) and report data (DD10)), while one other would very likely also to be of significance (raw sensor data (DD1), which might be used to calibrate specific cameras or camera models, etc.).

It is apparent that an adapted data type model for media forensics would be required to be able to make use of the full potential of the DCEA in this context. Nevertheless, it is outside the scope of this paper to propose such an adapted data type model.

## 4. Implementation of the Individual Detectors and the Fusion Operators

**Figure 1:**
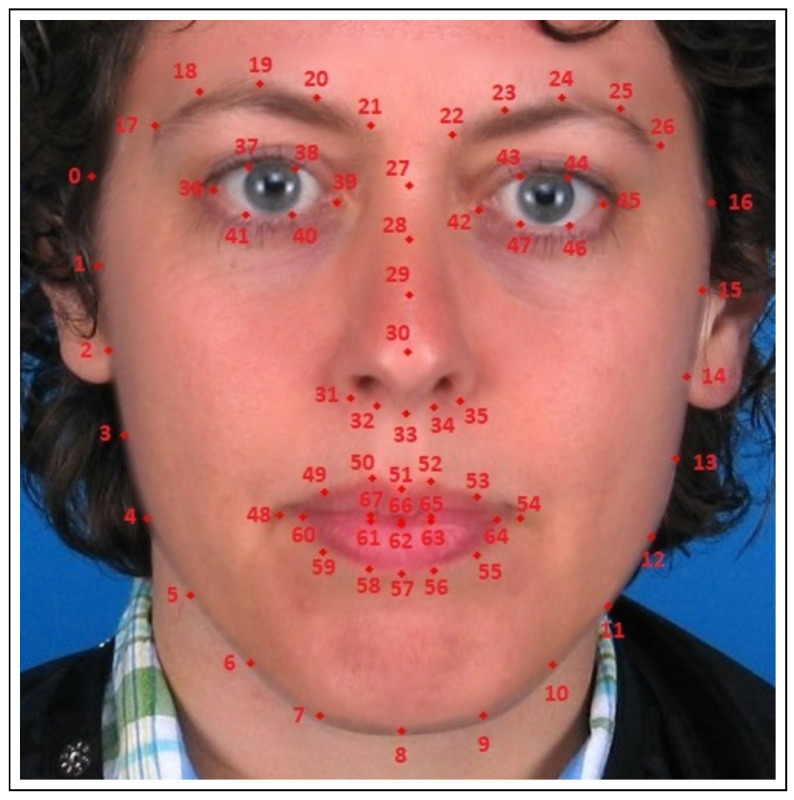
Visual representation of the 68 facial landmark model [35]. Image originates from Utrecht ECVP [37] with application of keypoints generation by dlib [36] followed by cropping.

For our DeepFake detection methods, the input video is evaluated frame-wise with the intention to analyze inter-frame patterns (e.g., the time between two blinks of one eye). In a pre-processing step, the presence of a face in a frame is determined, the face region is segmented and annotated frame-wise with a semantic model localizing 68 facial landmarks. This semantic model [35] is provided by the dlib library [36]. The output of this pre-processing is shown in Figure 1.

**Figure 2:**
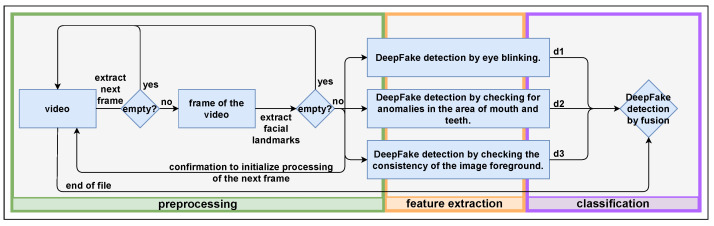
Concept pipeline considered in this paper.

In case no face can be localized in a frame, this event is logged, if a face is found, and the segmented face pixel matrix and the positions of these 68 facial landmarks are then forwarded to the feature extraction component of each individual detector as well as the concatenation operator for the feature-level fusion. This process is repeated frame-wise until the end of the video is reached, which initializes the detection operations performed. The entire processing sequence is shown in Figure 2. Due to the specific recording conditions of the datasets used in this paper (which all represent a single person in an ideal interview-like recording setting with perfectly illuminated faces and none of the facial key regions, such as eye and mouth, occluded), the pre-processing could be kept that simple. In case more realistic/averse videos have to be analyzed, this pre-processing would necessarily have to be extended.

The domain knowledge used here in hand-crafting features for DeepFake detection is based on the fact that DeepFake generators (similar to face morphing algorithms) rely on blending operations in the face region, which is a well established fact in the state-of-the-art research in this field [13]. Blending itself describes the process of a weighted combination of two or more faces to create a new identity [38]. This often goes hand in hand with a loss of local details in the face regions, while the background of a video or image is usually not affected, which is a fact also used in similar media forensics detectors such as, e.g., morphing attack detectors [39].

This knowledge is translated in Section 4.1 into three distinct hand-crafted feature spaces aiming at solving the following pattern recognition tasks to distinguish between DeepFake and genuine videos: (a) anomaly detection for eye blinking (Section 4.1.1), (b) anomaly detection in mouth and teeth region level of detail (Section 4.1.3), and (c) DeepFake detection based on image foreground texture (Section 4.1.3). In terms of the DCEA data type model, these features would make up the Trace characteristic feature data (DD5) from the data model discussed in [7] for digitized forensics. While the broad category actually fits, the extensive discussion on feature space design alternatives for DeepFake detection presented in Section 2.2 indicates that more detailed modeling would be required to sufficiently address this aspect.

To implement the actual classification, we decided not to design or implement our own but instead rely on a proven classification algorithm detection which does facilitate feature space as well as model-driven plausibility considerations. The actual algorithm that we use here is the WEKAs [40] J48 decision tree, which is an open source implementation of Ross Quinlans C4.5 decision tree algorithm [41]. The classifier is used here in its default parameterization, i.e., without parameter optimization being applied.

To further increase the performance and robustness of DeepFake detection, different fusion operators for feature-level fusion and decision-level fusion are implemented, as shown in Section 4.2.

In terms of datasets (i.e., processed signal data (DD2)), the pre-existing, publicly available and widely accepted reference datasets TIMIT-DF [16,42], FaceForensics++ [12,43,44] and Celeb-DF [13] are used in our evaluations. VidTIMIT [42], which was used to create TIMIT-DF [16], is a long-established reference database for various video processing tasks. It represents recording criteria that are ideal for face recognition and similar tasks: uniform lighting, the presence of exactly one person in each video, a frontal position to the camera, an average duration of 3 to 5 s and the speaking of ten different, pre-defined sentences. A total of 430 videos are included in the set, recorded using 43 volunteers. The resulting DeepFake videos were generated for TIMIT-DF by face swapping in two different resolutions with the autoencoder resolutions 64 × 64 and 128 × 128, respectively. Through prior selection, 16 suitable pairs of faces were selected for the generation, resulting in 32 DeepFake entities. This yields a total of 640 DeepFakes, which were taken into account in the TIMIT-DF dataset [16].

The second dataset considered is called DeepFakeDetection (DFD) [44], which originates from the FaceForensics++ [12] dataset. It contains a total of 363 source videos based on 28 actors (*DFD-source*). DeepFake synthesis was performed with an autoencoder resolution of 256 × 256 pixels and a total of 3068 DeepFake videos (*DFD-DF*) were generated. All videos considered were compressed with H.264 at CRF 23. Due to time constraints, only a subset of the DFD dataset, containing 55 DFD-source and 55 DFD-DF videos, were used. Video selection was carried out manually, selecting videos in which only a single person can be found speaking towards the camera. In the DFD dataset, this was carried out by searching for the keyword *talking* in conjunction with *against wall* or *outside*.

The third dataset is Celeb-DF [13], which includes videos (harvested from YouTube) of celebrities being interviewed. These source videos were divided in [13] into the two datasets *Celeb-YouTube* and *Celeb-real*, whereby only *Celeb-real* was considered for the DeepFake synthesis. The synthesis method is more advanced than the one from TIMIT-DF in terms of quality, using an autoencoder resolution of 256 × 256. Due to an average video duration of about 13 to 15 s, only a subset of this dataset is used in our own paper. For our evaluations, 120 source and 120 DeepFake videos were chosen. For simplification, the entire dataset is subsequently also referred to as *Celeb-DF*.

Those three datasets, summarized in Table 5 were used to design different training and testing scenarios to be able to establish facts about the generalization power of the detectors trained, which is an important aspect of the quality assessment for every method. Such evaluations would have to be performed as part of quality assurance in the strategic preparation (SP) phase of each forensic process.

### 4.1. Individual Detectors Using Hand-Crafted Features

In general, the 68 facial landmark model [35] used in this paper (see Section 4) can be structured into different facial areas, as shown in Figure 1. Here, the following segmentation alternatives are used to derive the features for our individual detectors: The first set of keypoints, numbers 0 to 26, describes the edges of the face along the chin and eyebrows. These keypoints are used to segment the image foreground, as explained in Section 4.1.3. Keypoints 27 to 35 describe the nose, which is neglected in this work. The eyes are described with the help of keypoints 36 to 47 and form the basis for the detection of blinking behavior considered in Section 4.1.1. The final keypoints, 48 to 67, are used to model the mouth, which is examined in more detail in Section 4.1.2.

In the following subsections, our three distinct detectors relying on different hand-crafted features spaces are described. A summarizing overview over all features extracted is presented in Table A1 at the end of the document in Appendix A.

#### 4.1.1. DeepFake Detection Based on Eye Blinking

The first implemented detector is based on the biometric modality eye and acts on the behavior of eye blinking. Using the 68 facial landmark model [35], each eye is described by six keypoints (keypoints 36 to 41 and 42 to 47, respectively). The process of blinking itself occurs subconsciously about 10 to 15 times per minute. On average one blink takes 0.3 to 0.4 s between closing and reopening the eyes. It should be noted that blinking behavior is also influenced by gender, age, time of day and how tired the person is [24]. In some publications, the minimum duration of human blinking is noted as 0.1 s [45]. To enable the detection of blinking, the eyes are modeled to two possible states—*open* and *closed*. To distinguish between these two states, the degree of aperture for each eye is determined individually by the formula:

$$AspectRatio=\frac{y_{Max}-y_{Min}}{x_{Max}-x_{Min}}$$

**Figure 3:**
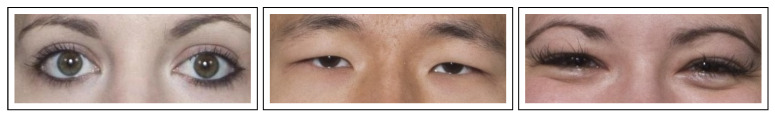
Illustration of the challenges of correctly detecting the aperture of eye opening as widely open (**left**), based on ethnicity (**center**) and inclusion of emotions (**right**). Images originate from LondonDB [46] dataset with application of cropping.

The parameters of this bounding box are determined from the six keypoints of the 68 facial landmark model, which describe the respective eye. The main idea of the feature design here is strong likeliness of DeepFake synthesis artifacts leading to lower average AspectRatio values, due to the inherent impact of the blending operation. Considering diversity in eye shapes and the inclusion of emotions, as shown in Figure 3, results on the use of a dynamic threshold (determined empirically on the training data used) were used to distinguish the eye states.

The eye state classification is carried out as binary decision, under the assumption that the aspect ratio always represents exactly one of two values, representing the two eye states (open and closed). The threshold under consideration was implemented as a bimodal distance function. Here, both states are described by a value which corresponds to the most frequent value of the upper and lower thirds of values found in the training data. The *closed* state is described by the most frequent value of the lower third of the value range. Conversely, *open* is described by the most frequent value, which is found in the upper third of the value range. Subsequently, the state for each eye and frame is determined via smaller distance to one of the two values representing the states.

For DeepFake classification based on eye blinking, a feature vector of fixed size of 13 dimensions was designed. Seven out of these 13 features are directly based on the AspectRatio, one is based on the difference between the two eyes and the other six are based on eyelid movements. This eyelid movement is detected as a rate of change on a frame-by-frame basis. Features 8 to 13 are based on the given eye state modeling. One feature introduces a new metric of anomaly, hereinafter referred to as *noise*. This noise is described as a frequent change in eye states below the expected frequency. In detail, this timespan is set to 0.05 s and thus corresponds to half the duration of a blink to detect anomalies only. Another feature describes the percentage of time in the video that the person has their eyes open. The last four features considered refer to the extreme values given the duration in each eye state.

In the summarizing overview of all features in this paper, given in Table A1 at the end of the document in Appendix A, these eye blinking features are the first 13 feature vector elements.

#### 4.1.2. DeepFake Detection Based on Mouth Region

**Figure 4:**
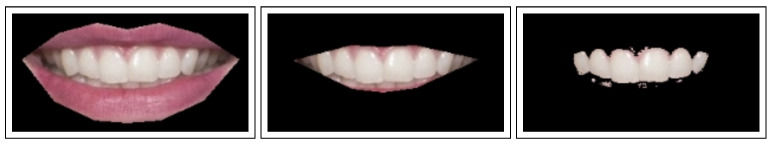
Illustration of the proposed representations for the mouth region OBR (**left**), IBR (**center**) and TR (**right**). Mouth image originates from LondonDB [46] dataset with application of keypoint generation by dlib [36], segmentation and cropping.

The second implemented detector is based on the biometric modality lip-movement. The focus of this approach is on analyzing the highly detailed teeth region. Under the assumption of blending as part of DeepFake creation, a blurred, less detailed image of the teeth is expected. The 68 facial landmark model is also used to localize the mouth region by using keypoints 48 to 67. These keypoints allow the mouth to be displayed as two separate images, one of which represents the entire mouth described by keypoints 48 to 59. This representation is henceforth called the OuterBoundRegion (OBR). The other keypoints (60 to 67) can be used show another representation considered in this work. This, in the following, is called the InnerBoundRegion (IBR) and represents the mouth area with the exception of the lips. The IBR is used to determine whether the mouth is open, since a closed mouth can be represented by a non-existent IBR. The third and last representation considered to describe the mouth region is the so-called TeethRegion (TR). The TR is created by segmenting the OBR to preserve potential teeth found in the image. An example of the representations can be found in Figure 4. In addition, the degree of aperture of the mouth is determined as an additional parameter based on the OBR. Here, the *x* and *y* dimensions are considered separately in order to act independently of the spoken phoneme. The respective values are determined by the bounding box of the OBR using $Aperture_{x}=x_{Max}-x_{Min}$ and $Aperture_{y}=y_{Max}-y_{Min}$ for each frame.

**Figure 5:**
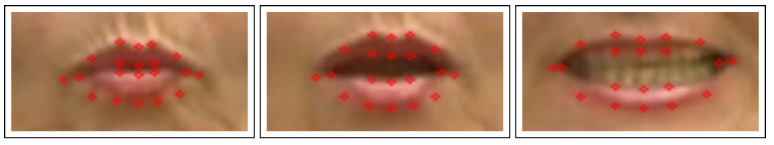
Illustration of the proposed mouth states: closed (**left**), open without detectable teeth (**center**) and open with detectable teeth (**right**). Image originates from VidTIMIT [42] dataset with application of keypoint generation by dlib [36] and cropping.

Based on these representations, a total of three states are conceived to describe the mouth. These states are: *closed mouth*, *open mouth without detectable teeth* and *open mouth with detectable teeth*. The subdivision of the states is made by two binary decisions. The first decision is based on the IBR and describes whether the mouth is open. The metric used for the decision is the number of pixels found in the IBR. Here, a conscious decision is made against cropping and scaling of the representations in order to prevent distortion of the image when viewing different visemes [47]. As a consequence, the number of pixels of the OBR is taken as a reference. Thus, the decision threshold is determined empirically on training data as: $\frac{PixelCount_{IBR}}{PixelCount_{OBR}}>0.211137$, for criteria for an open mouth. The second decision, if the mouth is classified as open, is made with the help of the number of pixels in the TR, once again using the OBR as a reference. The threshold considered (after empirical determination from training data) is $\frac{PixelCount_{TR}}{PixelCount_{OBR}}>0.11455$ for detectable teeth. An example of each state considered can be found in Figure 5.

For the detection of DeepFakes based on mouth region, a feature vector of dimensionality 16 is designed. Six of these features are based on mouth movements. This mouth movement is recognized image-wise as the rate of change, which corresponds to the extreme values for the *x*- and *y*-dimensions, respectively. The other 10 features are based on the detected mouth states, leaving out the *closed mouth* state. Thus, the focus of this review is based on the description of the level of detail in the mouth region. For this purpose, FAST and SIFT keypoint detectors as well as Sobel edge detection and the number of closed regions are considered. All of them are implemented by OpenCV [48] and used with default parameters. For the *open without teeth* state, the maximum of each feature, and for state *open with teeth*, the minimum of each feature are determined over all frames. Lastly, the percentage of time in both states is considered. The expectation for this approach is a low level of detail in the *open with teeth* state for DeepFakes or even a wrong assignment to the *open without teeth* state, although teeth are recognisable, due to blending of artifacts.

In the summarizing overview over all features in this paper, given in Table A1 at the end of the document in Appendix A, these mouth movement features are elements 14 to 29 of the feature vector.

#### 4.1.3. DeepFake Detection Based on Image Foreground

**Figure 6:**
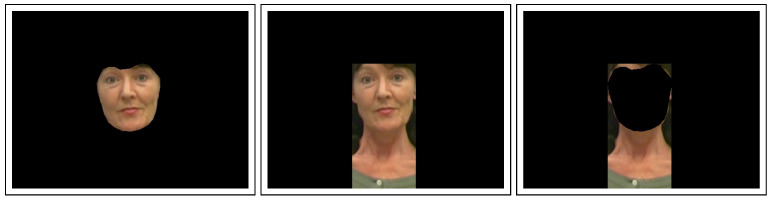
Illustration of the proposed representations for anomaly detection based on image foreground Img_Face_ (**right**). Image originates from VidTIMIT [42] dataset with application of keypoint generation by dlib [36] and segmentation.

The third and last proposed detector is based on domain transfer of hand-crafted features from a similar media forensics task. As shown by Kraetzer et al. [39], such a domain transfer seems plausible to detect blending anomalies in face morph attack detection. This requires an image foreground, which is characterized by a uniform distance towards the camera. Image foreground $Img_{Foreground}$ is designed as an extension of the facial region $Img_{Face}$, which is determined based on the 68 facial landmark model—more precisely, keypoints 0 to 26. The extension of the facial region is carried out by widening along the vertical axis to include the upper body, which is potentially shown in the image. A third representation, called $Img_{ROI}$, is conceived as the differential image of the previous two, formally described as $Img_{ROI}=Img_{Foreground}-Img_{Face}$. A visual example of each representation can be found in Figure 6.

For the detection of DeepFakes based on the image foreground, a feature vector of fixed size of eight elements was designed. The first subset of features is based on face detection itself, counting the number of frames and sequences where no face can be found. Here, it is assumed that a failure is due to anomalies of the DeepFake synthesis. The second set of features is based on the level of detail in $Img_{Face}$ relative to $Img_{ROI}$. For each frame and representation, the characteristics of FAST and SIFT keypoints as well as the Sobel edge image are determined. The implementation of these metrics is carried out using the default parameters given by OpenCV [48] and the scoring for each frame corresponds to $\frac{Img_{Face}}{Img_{ROI}}$. Lower values for DeepFakes are expected here. Lastly, the respective extreme values of all frames are extracted as features.

In the summarizing overview of all the features in this paper, given in Table A1 at the end of the document in Appendix A, these features are elements 30 to 37 of the feature vector.

### 4.2. Fusion Operators

To further increase the performance as well as robustness of the detection, different methods of fusion were implemented for our evaluation. The fusion itself is considered here both at *feature level* and *decision level* [49]. At the feature level, the feature spaces of the individual detectors are concatenated, without additional pre-processing such as weighting or filtering. At the decision level, a total of four operators are applied: The first operator makes an unbiased decision using *simple majority voting* [50]. In contrast, the other three operators implement weighted linear combinations and derive the weights for each individual detector based on its classification performance on the training set. Considering the different training scenarios, there are two sets of weights, each based on the training using dataset TIMIT-DF [16,42], DFD ( [12,44]) or Celeb-DF [13]. The explicit weights determined this way can be found in Section 5.2. In summary, the following five fusion operators are considered:

1. Feature-level fusion: concatenation of all features;
2. Decision-level fusion: simple majority voting;
3. Decision-level fusion: weighted, based on accuracy using TIMIT-DF for training;
4. Decision-level fusion: weighted, based on accuracy using DFD for training;
5. Decision-level fusion: weighted, based on accuracy using Celeb-DF for training.

## 5. Evaluation Results

The evaluation of the created approaches (i.e., our three feature spaces used in training and testing with the used J48 classifier) for DeepFake detection is looking into aspects of *performance*, *generalizability* and *plausibility* of the decisions made (i.e., the kind of information summarized in the DCEA data type model for digitized forensics as Classification result data (DD8)). To address *performance* and *generalizability*, the three datasets used for training and testing are presented as different scenarios (as shown in Table 6). Scenarios S1, S5 and S9, which represent evaluations in a simplistic (i.e., very naive) setup, split one dataset in disjointed training and test subsets. These three scenarios are used to validate the *performance* of detectors under optimal conditions.

In contrast, for evaluations on the *generalizability*, separate training and testing datasets are used in scenarios S2, S3, S4, S6, S7 and S8. Since the individual detectors classify binary according to *{DeepFake, OK}*, the evaluation is carried out using the metrics’ true positive rate (TPR; a true positive (TP) in our case being a DeepFake detected as a *DeepFake*), true negative rate (TNR; a true negative (TN) being an unmodified video classified as *OK*), accuracy and Cohen’s kappa (κ).

In addition, the hand-crafted features are evaluated in terms of *interpretability* and *relevance*. This is carried out by manually evaluating the trained decision trees in model-driven decision validation, looking at the individual features used to make the decision, the threshold used, and their distance from the root node. To support this analysis, the complete list of all features and experts’ assumptions about their content behavior can be found in Table A1 at the end of the document in Appendix A. To extend the initial model-driven decision validation, a comparison of the three decision trees trained on the different datasets, TIMIT-DF, DFD and Celeb-DF, is made.

### 5.1. Results for Individual Detectors

The detection approach based on *blink behavior* has a generally higher TPR than TNR, regardless of the scenario considered. For S1, it has a TNR of 70.47% and a TPR of 90.94%, resulting in an accuracy of 82.15% and κ of 0.6306. In comparison, S9 shows a TNR of 63.33% and TPR of 75.00%, resulting in an accuracy of 69.17% and κ of 0.3833. It is assumed that the Celeb-DF dataset also represents an improvement of the DeepFake synthesis over the older TIMIT-DF by incorporating more realistic blinking behavior. Considering the generalizability, a drastic decrease in detection rates can be seen in S7, S8 and S9, with a tendency to label all videos as DeepFake. In numbers, S3 indicates a TNR of 33.33% and TPR of 75.00%, with an accuracy of 54.17% and κ of 0.0833. In comparison, S7 shows a TNR of 6.05% and TPR of 99.53%, resulting in an accuracy of 61.96% and κ of 0.0659. By performing feature selection on the 13 features considered, only the eyelid movement-based features (ID2_blink_ to ID7_blink_) seem suitable. In addition, looking at the two eyes separately shows added value. As a result of the model-driven comparison of both trained decision trees, a DeepFake can be described by a higher difference between opening and closing speeds, relative to a non-manipulated video. However, the ranges of the values found as well as the associated thresholds are different for the TIMIT-DF and Celeb-DF datasets, explaining the drastic performance decrease for S3 and S7. Training on the DFD dataset shows only the use of features ID9_blink_ and ID10_blink_ for decision making.

The second detection approach considered, based on the *mouth region*, has the highest individual classification performances. For S1, a TNR of 88.84%, TPR of 97.81%, accuracy of 94.21% and κ of 0.8779 was achieved. In contrast, S9 resulted in a TNR of 91.67%, TPR of 97.50%, accuracy of 94.58% and κ of 0.8917, thus showing better results in direct comparison. Based on this result, it is suspected that newer DeepFake generators, such as the one used to create Celeb-DF, also exhibit said blending artifacts. Once again, there are clear losses in generalizability for S3 and S7: For S3, a TNR of 40.83%, TPR of 72.50%, accuracy of 56.67% and κ of 0.1333 were observed. S7 shows slightly better results with a TNR of 63.02%, TPR of 71.09%, accuracy of 67.85% and κ of 0.3378, which are justified by more general inclusion conditions of the Celeb-DF data and more general classification model. Based on the 16 features considered in feature selection, the set of features describing the grade of detail, excluding the ones using Sobel operator, are used to classify a DeepFake. This clearly shows that blending results in a loss of detail in the facial region, which can be found for both states *open without teeth* and *open with teeth*. Additionally, the assumption that the state *open with teeth* is found less frequently for DeepFakes is correct. However, it should be noted here that the approach only works if an open mouth can be found—for example, if a person is speaking.

The trend of high TPR at the expense of TNR is also emerging for the *detector based on the image foreground*. For S1, a TNR of 52.33%, TPR of 87.50%, accuracy of 73.36% and κ of 0.4182 were observed. For S9, the results look similar, with a TNR of 56.67%, TPR of 85.00%, accuracy of 70.83% and κ of 0.4167. This approach also shows poor generalizability, with a TNR of 43.33%, TPR of 70.00%, accuracy of 56.67% and κ of 0.1333 for S3. Lastly, S7 shows a TNR of 32.79%, TPR of 79.38%, accuracy of 60.83% and κ of 0.1297. For the decision making itself, the features based on the level of detail except for the Sobel operator, as well as the number of frames without a found face, are used. However, the ID1_foreground_ shows a different classification strategy depending on the dataset considered, when at least one frame without a face is found. While for TIMIT-DF and DFD it is interpreted as a DeepFake, for Celeb-DF it serves the classification OK. It is suspected that for TIMIT-DF and DFD, the synthesis may result in artifacts, making the face undetectable. On the other hand, less strict recording conditions in Celeb-DF do not exclude side shots that cannot be detected by the facial landmark model. The use of features ID3_foreground_ to ID6_foreground_ corresponds to the assumptions about blending, whereby lower levels of detail are taken as an indication of a DeepFake.

In conclusion, regardless of the detection approach considered, in all cases, a value for Cohen’s kappa >0 was obtained, implying for all cases a detector performance better than chance agreement (i.e., better than guessing). Nevertheless, it has to be admitted that the differences between the more naive setups (S1 and S9 with κ>0.35) and the more realistic setups (S3 and S7 with κ<0.15 for all but one case) indicate a very limited generalization power of the trained detectors.

Analyzing the trained models in more detail, it has to be highlighted that the decision tree trained on Celeb-DF is shown to be smaller and more compact. This is justified by a lower number of suitable features for the detection of higher quality DeepFakes. In addition, S3 generalizes better than S7, which goes hand in hand with the preceding statement. Here, Celeb-DF represents a more general dataset, with fewer indicators of DeepFakes, where the trained model applies better to TIMIT-DF than vice versa.

### 5.2. Results for Fusion Operators

**Figure 7:**
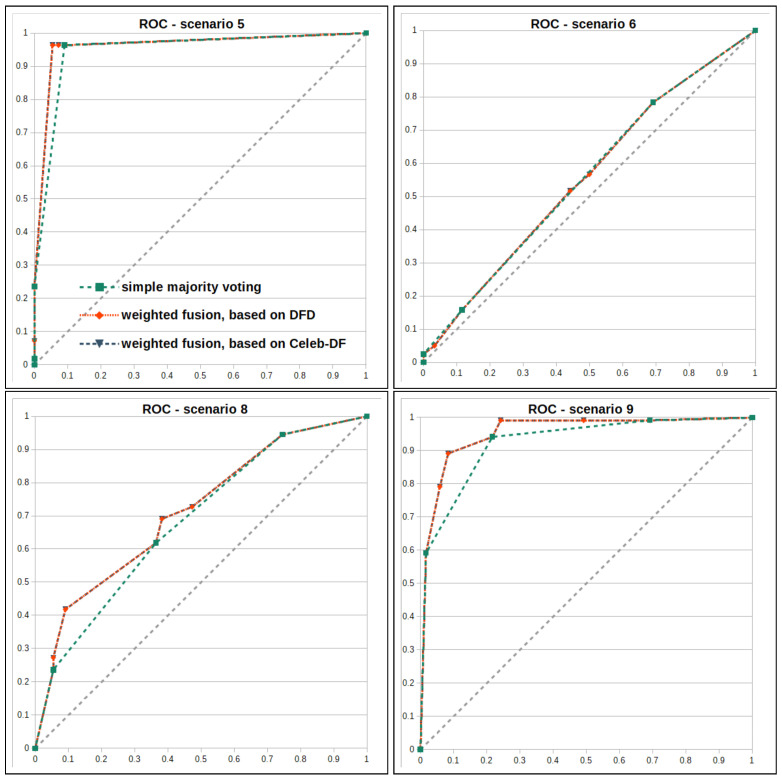
Receiver operation curves (ROCs) for the decision-level fusion methods simple majority voting and weighted fusion, based on DFD and Celeb-DF. Scenarios S5, S6, S8 and S9, which consider both the DFD and Celeb-DF datasets, are presented here. The false alarm rate (false positive rate) is plotted on the *x*-axis. The sensitivity (true positive rate) is plotted on the *y*-axis.

For all fusion operators considered, the metrics TPR, TNR, accuracy and Cohen’s kappa are used to allow comparability between fusion and individual detectors. In addition, the *receiver operating characteristic* (ROC) for all scenarios considered, based on the different approaches of fusion at the decision level, are determined. The resulting graphs can be found in Figure 7. Based on the ROC, the *area under curve* (AUC) is determined in order to realize a better comparison with research results in the state of the art in the literature.

The first fusion approach considered is carried out at the *feature level* by concatenating all features without prior adjustments or filtering. A descriptor of this vector can be found in Table A1. For S1, a TNR of 96.74%, TPR of 98.13%, accuracy of 97.57% and κ of 0.9494 and for S9 a TNR of 92.50%, TPR of 95.83%, accuracy of 94.17% and κ of 0.8833 are achieved. This outperforms the best individual detector from Section 5.1. However, this performance is accompanied by even more significant losses for generalizability seen for S3 and S7: A TNR of 70.83%, TPR of 38.33%, accuracy of 54.58% and κ of 0.0917 are achieved for S3 and a TNR of 63.02%, TPR of 64.22%, accuracy of 63.74% and κ of 0.2653 are achieved for S7.

The model-driven feature selection shows that mainly features of the mouth region are used here. From the other two feature spaces, only ID2_blink_ and ID6_foreground_ are considered (the latter is found in the root of the respective decision trees). This again implies that the individual features based on blinking and image foreground appear more unsuitable than the features based on the mouth region. In addition, the differences between the performances on the datasets and corresponding differences in threshold determination described at the end of Section 5.1 are again apparent.

The second approach of the fusion operators takes place at *decision-level* in the form of *simple majority voting*. Here, detection rates of TNR of 79.53%, TPR of 98.75%, accuracy of 91.03% and κ of 0.8075 for S1 and TNR of 78.33%, TPR of 94.17%, accuracy of 86.25% and κ of 0.7250 for S9 are determined. Furthermore, simple majority voting shows the best generalizability of all approaches for S3, with a TNR of 53.33%, TPR of 64.17%, accuracy of 58.75% and κ of 0.1750. A TNR of 26.74%, TPR of 91.41%, accuracy of 65.42% and κ of 0.2015 are determined for S7.

For the considered weighted *decision-level* fusion approaches, the weight combinations w_blink_ = 0.328967, w_mouth_ = 0.377246 and w_foreground_ = 0.293787 based on the use of TIMIT-DF for training, w_blink_ = 0.257934, w_mouth_ = 0.420621 and w_foreground_ = 0.321445 based on the use of DFD as well as w_blink_ = 0.294849, w_mouth_ = 0.403197 and w_foreground_ = 0.301954 based on the use of Celeb-DF for training are derived based on the determined detection performances in training. In addition, the optimal threshold value for the classification is determined manually. For both cases, the ideal threshold can be described as:

$$w_{blink}+w_{foreground}<threshold<w_{mouth}+w_{blink|foreground}$$

It is therefore necessary that both the detector based on the mouth region and another one arrive at the classification result *DeepFake* so that the fusion also arrives at that conclusion. In the following, a threshold value of 0.65 is used. Considering the results, these resemble the detector based on the mouth region and show S1 with a TNR of 91.40%, TPR of 97.03%, accuracy of 94.77% and κ of 0.8904, as well as a TNR of 91.67%, TPR of 94.17%, accuracy of 90.42% and κ of 0.8083 for S9. In the context of generalizability, this fusion approach for S3 shows a TNR of 59.17%, TPR of 55.83%, accuracy of 57.50% and κ of 0.1500. Scenario S7 has a TNR of 63.72%, TPR of 70.94%, accuracy of 68.04% and κ of 0.3427 are determined, representing the best results of all considered implementations for S7. A marginal improvement of the weights based on the Celeb-DF can be found in consideration of the ROC AUC, as shown in Figure 7.

In conclusion, previous trends are confirmed showing that S7 has a higher performance than S3 and thus more refined DeepFakes and less limiting factors of acquisition are necessary for a more accurate classifier.

Table 7 summarizes and compares the performances of the individual and fusion detectors. While the best performances are very similar, the fusion-based approaches show a much smaller range in their results, which implies that the strongest of the three single detectors (using the mouth region features) has a dominating impact out of all three fusion operators tested. By switching from single classifiers to fusion approaches, here no gain could be made in terms of increasing generalization power. The reason has to be sought in the different thresholds that were derived for both training sets (see the corresponding discussion at the end Section 5.1).

## 6. Summary and Conclusions

To allow for a direct comparison of hand-crafted and learned features, Section 6.1 discusses our obtained performances and the generalization behavior observed in direct comparison with a state-of-the-art paper using deep learning under comparable evaluation conditions. Furthermore, we compare our feature concept implementations for *eye blinking*, *mouth region* and *foreground texture analysis* with other hand-crafted and learned features considering the same facial regions.

In Section 6.2, we summarize our conclusions on the comparison of hand-crafted and learned features for DeepFake detection.

### 6.1. Summary of the Results and Comparison with other Approaches from the State of the Art

In the sections below, we provide a comparison of the results obtained in our experiments with selected work from the state of the art in this fast growing research field. Section 2.1 shows that there exists a wide range of different approaches to distinguish DeepFake from real videos, with a strong tendency towards relying on features learned by using neural networks. In subSection 6.1.1, we compare our results with selected detection performances and generalization behaviors observed in the state of the art. In Section 6.1.2, we compare our concepts for feature designs (looking at hand-crafted features, especially for eye blinking, mouth region and image foreground) with similar approaches by other authors.

#### 6.1.1. Performances and Generalization Power

Table 8 consists of two parts, the upper half represents our results on fusion-based detectors trained on the DFD and Celeb-DF dataset and tested on TIMIT-DF, DFD and Celeb-DF. The values given above are the results taken from Table 7 translated into area under curve (AUC).

The second half are the results resented by Bondi et al. in [9], where the authors performed very similar experiments like us only with a feature space learned with a convolutional neural network (CNN). In their paper, they also used a total of four sets to design training and test setups as we did with our S1 to S9. Two of the sets are Celeb-DF and DFD, which are also used by us. Comparing our work and the AUC results from Bondi et al. on the sets that are used in both papers, we can state that our approach with hand-crafted features performs only slightly worse (maximum AUC = 0.960) than their method relying on learned features (maximum AUC = 0.998). Furthermore, we can point out that their experiments with training and testing on different sets of DeepFakes results in very similar, if not worse problems in terms of generalization power (i.e., AUC drops from values larger than 0.9 to smaller than 0.7).

#### 6.1.2. Comparison of Feature Concepts

In the case of DeepFake detection, *eye blinking* is a feature which is used for hand-crafted as well as learned feature space approaches. Section 2.1 also recaps the main functionality of DeepVision by Jung et al. [24] where they describe a hand-crafted detection method of the eye blinking behavior of persons in potential DeepFake videos. This approach is similar to our proposed feature detector for the eye blinking behavior. After the face detection happens in both cases, the detection of both eyes frame-by-frame. In our work, for every detected eye the AspectRatio changes are tracked over time. Jung et al. [24] evaluate only the amount of blinking events in a video and also the blink elapsed time as well as the blinking period time, which would correspond to the features ID8_blink_ to ID13_blink_ of our work. Implementation differences are visible in handling the threshold for state (open vs. closed) determination.

Li et al. [15] used a CNN for the segmentation of the eyes after they located the face area in a video. For their inter-frame blinking analysis they use an RNN with LSTM cells. The output of each RNN neuron is connected to a fully connected network, which estimate the output of the LSTM cells if an eye is open or closed.

Unfortunately, a direct comparison with these other publications in terms of performances is not possible here, since entirely different datasets were used.

To our knowledge, there is currently in the literature no similar DeepFake detection approach analyzing only the visible *mouth region* in the video with hand-crafted features. Currently, our approach only analyzes the mouth region in the video stream but does not consider of the spoken speech in the audio stream combined with the lip movements. Extending it with methods for fake voice detection, as in [51], would be an interesting next step for this method.

Considering neural network-based approaches for analysing the mouth region, Agarwal et al. [47] present the hypothesis that DeepFake videos are not able to reproduce spoken phoneme such as "M", "B" or "P", where the mouth is normally completely closed for the pronunciation. Their detection pipeline starts with the extraction of all phoneme locations. The phoneme generation is managed by the transcribing API Speech-To-Text of Google and then manually reduced to six phoneme groups ({OY,UH,UW}, {AA}, {M,B,P}, {L}, {F,V}, {CH,JH,SH}). The video stream is then aligned to these phonemes. After that, they measure the visemes for several evaluation tests in three different ways (manual, profile, CNN) [47]. This approach corresponds to a simplified lip-sync approach for a DeepFake detection, which is realized in [16] (see Section 2.1).

To the best of our knowledge, in the current literature, no hand-crafted approach analyzing only the *image foreground* to detect DeepFakes using image foreground can be found.

Looking for neural network-based approaches implementing such a feature space, the papers of Zhang et al. [52,53] have to be mentioned here. In contrast to our approach, they developed an automatic approach using a CNN. The idea behind their approach is that the image compression ratio of the face and background is different between the DeepFake and original. The reason behind this issue is that the resolution all current DeepFake algorithms is very limited. In addition, the generated fake faces are modified by affine transformations such as scaling, rotating and shearing. Based on this hypothesis, Zhang et al. try to detect the resulting artifacts of these affine transformations. The detection of the compressing distortions happens in their case with the well known error level analysis (ELA) method [54]. It follows that the training of a CNN with these ELA images which extracts the counterfeit features of the ELA images. If the CNN is able to extract these counterfeit features, then the input image of the CNN is a DeepFake. Even though the detection in [52,53] uses only DeepFake images in its tests, it would be possible to upgrade this approach for a DeepFake detection of videos.

### 6.2. Comparison of Hand-Crafted and Learned Features for DeepFake Detection and Conclusions

Our proposed hand-crafted features as well as hand-crafted features from other sources such as [21,22,23,24] have shown that also such expert knowledge-driven approaches are able to distinguish real from DeepFake videos. The detection rates are usually high but in most cases slightly lower than the performance achieved with learned feature spaces. The main advantage that hand-crafted features have over learned features is their interpretability and the consequences this might have for plausibility validation for decisions made.

All current approaches for DeepFake detection in the literature show error rates which are far from perfect. In particular, when DeepFake detectors are evaluated in a realistic setting, i.e., with independent training and test sets, then current hand-crafted as well as learned feature space approaches suffer generalization problems if the characteristics of training and test data are different. This has been demonstrated in our results but also in papers performing similar tests with learned feature spaces, such as Bondi et al. in [9].

Obviously, the problems of individual detectors could be increased if the DeepFake generators would include active mechanisms (counter-forensics) into the generation process to enforce false results with known detectors. Various strategies could and should be applied to address these performance and reliability issues. In this paper, we performed fusion operations to improve detection performances of hand-crafted feature spaces. In their work, Lin et al. [29] propose to extend fusion even further by combining hand-crafted features and CNN features. By doing so, they imply that it would enable us to find a solution that combines the interpretability of hand-crafted features with the potentially higher classification accuracy of learned features. The main benefit of such fusion approaches is that they generate complexer decision constructs that could compensate the problems of individual detectors in the set and might be more resilient against counter-forensics. However, these benefits would be bought at the cost of throughput/runtime behavior and a much more difficult interpretability of decisions.

In most cases, hand-crafted approaches do not need much data for model training, which may also result in lower process costs for memory or calculation time. Additionally, approaches which are including neuronal networks and specially convolutional neuronal networks need much more memory (mostly graphic memory) and CPU or GPU power for the training of the detection networks. In particular, the analyzing process of whole videos and specially a recurrent network structure have a huge impact to the needed memory. These learned approaches are also expensive in purchase costs for (new) hardware architectures. However, when the networks are finally trained, the networks are able to detect DeepFake videos in a very short time, similar to models created/trained with hand-crafted features. Therefore, neither choice would limit the application in incident response procedures (EMID), where fast (close to real time) detector responses would be required for live detectors.

## 7. Future Work

Our proposed hand-crafted features reach acceptable detection rates for DeepFake videos. However, not every video was classified correctly. Some DeepFake videos were detected as real video and vice versa. It is necessary to detect, analyze and find the reasons for a misclassification to improve our proposed approaches for DeepFake detection. A further improvement can be achieved by investigating different feature selection methods to strengthen the suitability of the proposed features. Possible improvements would also affect approaches from other sources, as it is extremely unlikely that any detection method can correctly classify every video, especially considering potential counter-forensics methods included in the DeepFake generation. Different detection approaches should be analyzed and the benefits of these approaches should be finally combined into a single detection method with a better detection rate and higher robustness against counter-forensics. This also concerns the fusion of hand-crafted and learned features whereat also the integration of hand-crafted methods into learned approaches are meant. In this context, the evaluation of our approaches should expand to other DeepFake databases to create a wider base for training or construct more evaluation scenarios to validate the generalizability of the approach.

A DeepFake video usually consists of two media types: the visible video and the underlying audio. These different media types should be analyzed in combination at the same time. For example, our handcrafted detector for the mouth region should be expanded to include a lip synchronization detector. It is also possible to extract the current emotion of a person in a video. Here, it is imaginable to analyze the emotion of one area (e.g., the left eye) and compare it to another (e.g., the right eye and/or the mouth). Possible aspects to determine emotions are facial expression (e.g., gesture of mouth and eyes), as well as the way of speaking.

In this paper, we started with trying to project the media forensics method of DeepFake detection onto a forensic process model (here, the data-centric examination approach (DCEA) introduced in Section 2.3). In future work, more effort is required to extend this projection, including a required extension of the DCEA data type model to make it suitable for the media data characteristics encountered here. As discussed in Section 3, the most significant change would be the design of a new, domain specific data type model for this media forensics task. While many components (such as the Processed signal data (DD2), Contextual data (DD3), Classification result data (DD8), Chain of custody data (DD9) and Report data (DD10)) could be re-used with only minor modifications, others (esp. Parameter data (DD4), Trace characteristic feature data (DD5) as well as Model data (DD7)) would need a major overhaul. The updated data modeling would also have to reflect that, in this media forensics task, different correlated (media) data streams such as video, audio, network, meta and synchronization data would have to be analyzed in parallel to substantiate the findings.

In addition to the data-driven nature of DCEA, a second reason for its choice as a forensic process model here is that it explicitly requests of modeling the error, (information) loss and (decision) uncertainty of forensic methods [7]. These considerations have to by extended for media forensics from closed set tests (where the ground truth class label in a pattern recognition problem is known) to field applicability (where only the detector response is available and the true class of a specimen encountered will remain unknown).

## Figures and Tables

**Table 1:** Sets of examination steps for digital forensics, as defined in [7] (updated from [32]).

Sets of Examination Steps	Description (According to [7])
Strategic preparation (SP)	Includes measures taken by the operator of an IT system and by the forensic examiners in order to support a forensic investigation prior to an incident
Operational preparation (OP)	Includes measures of preparation for a forensic investigation after the detection of a suspected incident
Data gathering (DG)	Includes measures to acquire and secure digital evidence
Data investigation (DI)	Includes measures to evaluate and extract data for further investigation
Data analysis (DA)	Includes measures for detailed analysis and correlation between digital evidence from various sources
Documentation (DO)	Includes measures for the detailed documentation of the proceedings, also for the transformation into a different form of description for the report of the incident

**Table 2:** Forensic data types defined in [7] (updated from [34]).

Forensic Data Type	Description (According to [7])
Raw data	A sequence of bits or data streams of system components not (yet) classified
Hardware data	Data not or only in a limited way influenced by the OS and application
Details about data	Meta data describing other data
Configuration data	Modify the behavior of the system and applications
Communication protocol data	Modify the communication behavior of the system
Process data	Data about a running process
Session data	Data collected by a system during a session
User data	Content created, edited or consumed by the user

**Table 3:** Forensic data types defined in [7] for an exemplary selected process in digitized forensics (here, digital dactyloscopy) (updated from [32]).

Forensic Data Type	Description (According to [7])
Raw sensor data (DD1)	Digital input data from the digitalization process (e.g., scans of test samples)
Processed signal data (DD2)	Results of transformations to raw sensor data (e.g., visibility enhanced fingerprint pattern)
Contextual data (DD3)	Contain environmental data (e.g., spatial information, spatial relation between traces, temperature, humidity)
Parameter data (DD4)	Contain settings and other parameters used for acquisition, investigation and analysis
Trace characteristic feature data (DD5)	Describe trace specific investigation results (e.g., level 1/2/3 fingerprint features)
Substrate characteristic feature data (DD6)	Describe trace carrier specific investigation results (e.g., surface type, individual surface characteristics)
Model data (DD7)	Describe trained model data (e.g., surface specific scanner settings, reference data)
Classification result data (DD8)	Describes classification results gained by applying machine learning and comparable approaches
Chain of custody data (DD9)	Describe data used to ensure integrity and authenticity and process accompanying documentation (e.g., cryptographic hash sums, certificates, device identification, time stamps)
Report data (DD10)	Describe data for the process accompanying documentation and for the final report

**Table 4:** Grouping of sets of methods for the forensic process in digital forensics defined in [7] (updated from [32]).

Sets of Methods for the Forensic Process in Digital Forensics	Description (According to [7])
Operating system (OS)	Methods that provide forensically relevant data as well as serving their main purpose of distributing computing resources
File system (FS)	Methods that provide forensically relevant data as well as serving their main purpose of maintaining the file system
IT application (ITA)	Methods provided by IT applications that provide forensically relevant data as well as serving their main purpose
Explicit means of intrusion detection (EMID)	Methods that are executed autonomous on a routine basis and without a suspicion of an incident
Scaling of methods for evidence gathering (SMG)	Methods that are unsuited for routine usage in a production environment (e.g., due to false positives or high computation power requirements)
Data processing and evaluation (DPE)	Dedicated methods of the forensic process that display, process or document information

**Table 5:** Collection of datasets used for this paper.

Dataset	Number of Videos	Reference
VidTIMIT	430 *	[42]
TIMIT-DF	640	[16,42]
DFD-source	55 *	[12,44]
DFD-DF	55 *	[12,44]
Celeb-YouTube	60 *	[13]
Celeb-real	60 *	[13]
Celeb-DF (v2)	120 *	[13]

*: Numbers do not reflect the total but rather the number of videos used in the context of this work.

**Table 6:** Representation of the considered training and testing scenarios, given by differentiation of the training and testing datasets used.

↓ Training/Testing →	TIMIT-DF	DFD	Celeb-DF
TIMIT-DF	scenario 1 (S1)	scenario 2 (S2)	scenario 3 (S3)
DFD	scenario 4 (S4)	scenario 5 (S5)	scenario 6 (S6)
Celeb-DF	scenario 7 (S7)	scenario 8 (S8)	scenario 9 (S9)

**Table 7:** Classification results based on accuracy in percent, followed by Cohen’s kappa in parenthesis, for the different methods proposed in this paper. Best result for each combination of training and test data is highlighted bold.

Training Dataset →	TIMIT-DF [16,42]			DFD [12,44]			Celeb-DF [13]		
↓ proposed method test dataset →	TIMIT-DF	DFD	Celeb-DF	TIMIT-DF	DFD	Celeb-DF	TIMIT-DF	DFD	Celeb-DF
DeepFake detection based on	82.15%	50.00%	**57.50%**	58.32%	59.09%	52.92%	62.06%	58.18%	69.17%
eye blinking	(0.63)	(0.00)	**(0.15)**	(0.15)	(0.18)	(0.06)	(0.07)	(0.16)	(0.38)
DeepFake detection based on	**94.21%**	**76.36%**	56.67%	**64.95%**	**96.36%**	53.75%	**67.85%**	**69.09%**	**94.58%**
mouth region	**(0.88)**	**(0.53)**	(0.13)	**(0.29)**	**(0.93)**	(0.08)	**(0.34)**	**(0.38)**	**(0.89)**
DeepFake detection based on	73.36%	53.64%	56.67%	58.33%	73.64%	**54.02%**	60.83%	54.55%	70.83%
image foreground	(0.42)	(0.07)	(0.13)	(0.17)	(0.47)	**(0.11)**	(0.13)	(0.09)	(0.42)
Feature-level fusion	**97.57%**	66.36%	54.58%	65.05%	**97.27%**	**56.25%**	63.74%	60.00%	**94.17%**
	**(0.95)**	(0.33)	(0.09)	(0.30)	**(0.95)**	**(0.13)**	(0.27)	(0.20)	**(0.88)**
Decision-level fusion:	91.03%	69.09%	**58.75%**	59.72%	61.18%	52.08%	65.42%	62.73%	86.25%
simple majority voting	(0.81)	(0.38)	**(0.18)**	(0.24)	(0.24)	(0.04)	(0.20)	(0.25)	(0.73)
Decision-level fusion:	94.77%	**70.91%**	57.50%	**67.00%**	95.45%	53.75%	**68.04%**	**65.45%**	90.42%
weighted (threshold=0.65)	(0.89)	**(0.42)**	(0.15)	**(0.33)**	(0.91)	(0.08)	**(0.34)**	**(0.31)**	(0.81)

**Table 8:** Comparison (in terms of AUC) of different state-of-the-art DeepFake detectors with the presented methods. Further separation based on differentiating training and test dataset.

Training Dataset →	DeepFakeDetection (DFD) [12,44]			Celeb-DF [13]		
↓ fusion method test dataset →	TIMIT-DF	DFD	Celeb-DF	TIMIT-DF	DFD	Celeb-DF
Ours: simple majority	0.668	0.947	0.556	0.690	0.685	0.925
Ours: weighted based on accuracy	0.685	0.960	0.556	0.682	0.712	0.954
using DFD for training						
Ours: weighted based on accuracy	0.685	0.960	0.556	0.698	0.712	0.955
using Celeb-DF for training						
[9]: Baseline	-	0.987	0.754	-	0.708	0.998
[9]: Triplet Training	-	0.882	0.759	-	0.554	0.995
[9]: EfficientNetB4. Binary Cross
Entropy with augmentation	-	0.990	0.842	-	0.795	0.998
[9]: EfficientNetB4. Triplet Loss
with augmentation	-	0.982	0.809	-	0.604	0.995

## Data Availability

In this work, the following the pre-existing reference databases have been used for our evaluations: TIMIT-DF ([16,42]), Celeb-DF ([13]) and FaceForensics++ ([12,43,44]). They are publicly available at: https://www.idiap.ch/en/dataset/deepfaketimit (last accessed: 30 June 2021); https://github.com/yuezunli/celeb-deepfakeforensics (last accessed: 30 June 2021); https://github.com/ondyari/FaceForensics (last accessed: 30 June 2021).

## Appendix A. Collection of Features Proposed in this Paper

**Table A1:** Collection of all features and their expected behaviors proposed in this paper.

ID	Feature	Description
ID1_fusion_ ID1_blink_	Maximum AspectRatio difference between both eyes.	The expected difference is close to 0, whereby a larger distance is suspected as an indication of a DeepFake. Additionally, the absence of winking is required for this feature.
ID2_fusion_ ID2_blink_	Absolute maximum AspectRatio rate of change for the left eye.	Based on several studies the eyelid movement varies based on different aspects, e.g., age and gender [24,45]. Nevertheless, the maximum speeds, as well as the relation of opening and closing speeds, could be an indication for DeepFake detection. This rate of change for each frame is determined by the difference between previous and following frame. Normalization is carried out by multiplying the rate of change by the frame rate of the video. This results in the AspectRatio change every 3 seconds, described as $\frac{\Delta AspectRatio}{3s}$. The suitability of these features is based on the disregard of blink behavior in DeepFake synthesis.
ID3_fusion_ ID3_blink_	Maximum AspectRatio rate of change for the left eye. Maximum opening speed of the left eye.	
ID4_fusion_ ID4_blink_	Minimum AspectRatio rate of change for the left eye. Maximum closing speed of the left eye.	
ID5_fusion_ ID5_blink_	Absolute maximum AspectRatio rate of change for the right eye.	
ID6_fusion_ ID6_blink_	Maximum AspectRatio rate of change for the right eye. Maximum opening speed of the right eye.	
ID7_fusion_ ID7_blink_	Minimum AspectRatio rate of change for the right eye. Maximum closing speed of the right eye.	
ID8_fusion_ ID8_blink_	Noise count in the eye state signal.	*Noise* is defined as a rapid change of eye state, where one state lasts for a maximum of 0.08 seconds. A higher number of these noises is expected for DeepFakes.
ID9_fusion_ ID9_blink_	Percentage of video time at which the state *open* is classified.	Another feature that can be justified by studies about human blinking behavior [24,45]. Assuming a healthy person in a non-manipulated video, on average a value of about 0.9 should be expected.
ID10_fusion_ ID10_blink_	Minimum duration detected for the eye state *open* in seconds.	Features based on the durations of the states are again based on the knowledge of human blinking behavior. It is assumed that the eyes are open longer than they are closed. As a conclusion ID12_blink_ < ID10_blink_ and ID13_blink_ < ID11_blink_ are expected.
ID11_fusion_ ID11_blink_	Maximum duration detected for the eye state *open* in seconds.	
ID12_fusion_ ID12_blink_	Minimum duration detected for the eye state *closed* in seconds.	
ID13_fusion_ ID13_blink_	Maximum duration detected for the eye state *closed* in seconds.	
ID14_fusion_ ID1_mouth_	Absolute maximum rate of change in y-dimension.	This rate of change for each frame is determined by the difference between previous and following frame. Normalization is carried out by multiplying the rate of change by the frame rate of the video. This results in the AspectRatio change every 3 s, described as $\frac{\Delta AspectRatio}{3s}$. For these features, a maximum speed is assumed, which is determined by training the model. Exceeding this maximum speed is assumed to be an indication for the classification DeepFake. Limitation: only works with videos where the person moves their lips during the video, e.g., when speaking.
ID15_fusion_ ID2_mouth_	Maximum rate of change in y-dimension. Lip opening movement in y-dimension.	
ID16_fusion_ ID3_mouth_	Minimum rate of change in y-dimension. Lip closing movement in y-dimension.	
ID17_fusion_ ID4_mouth_	Absolute maximum rate of change in x-dimension.	
ID18_fusion_ ID5_mouth_	Maximum rate of change in x-dimension. Lip opening movement in x-dimension.	
ID19_fusion_ ID6_mouth_	Minimum rate of change in x-dimension. Lip closing movement in x-dimension.	
ID20_fusion_ ID7_mouth_	Percentage of video time at which the state *open without teeth* is classified.	The assumption for feature ID7_mouth_ is that DeepFakes are more often classified in this state compared to non-manipulated videos. The cause is the blending subprocess in the creation of DeepFakes, which leads to a loss of information and detail in the mouth region due to smoothing. As a consequence, DeepFakes are assumed to have both a comparatively low level of detail due to said blending and a comparatively high level of detail due to possible misclassification of *open with teeth* as *open without teeth*. Normalization takes place relative to the number of pixels in the TR (see Figure 4). Default value is set to -1 to be outside the considered range.
ID21_fusion_ ID8_mouth_	Maximum number of regions based on all frames of the video for state *open without teeth*.	
ID22_fusion_ ID9_mouth_	Maximum number of FAST keypoints based on all frames of the video for state *open without teeth*.	
ID23_fusion_ ID10_mouth_	Maximum number of SIFT keypoints based on all frames of the video for state *open without teeth*.	
ID24_fusion_ ID11_mouth_	Maximum number of Sobel edge pixels based on all frames of the video for state *open without teeth*.	
ID25_fusion_ ID12_mouth_	Percentage of video time at which the state *open with teeth* is classified.	The assumption for feature ID12_mouth_ is that non-manipulated videos are more often classified in this state compared to DeepFakes. The cause is the blending subprocess in the creation of DeepFakes, which leads to a loss of information and detail in the mouth region due to smoothing. As a consequence, DeepFakes are assumed to have a comparatively low level of detail due to said blending. Normalization takes place relative to the number of pixels in the TR (see Figure 4). Default value is set to −1 to be outside the considered range.
ID26_fusion_ ID13_mouth_	Minimum number of regions based on all frames of the video for state *open with teeth*.	
ID27_fusion_ ID14_mouth_	Minimum number of FAST keypoints based on all frames of the video for state *open with teeth*.	
ID28_fusion_ ID15_mouth_	Minimum number of SIFT keypoints based on all frames of the video for state *open with teeth*.	
ID29_fusion_ ID16_mouth_	Minimum number of Sobel edge pixels based on all frames of the video for state *open with teeth*.	
ID30_fusion_ ID1_foreground_	Total number of frames in the video without a detectable face.	The consideration of these features is made under the assumption that DeepFake synthesis could result in artifacts, causing the face detection to fail. Normalization is relative to the number of frames of the video to ensure comparability regardless of the video length.
ID31_fusion_ ID2_foreground_	Total number of segments in the video without a detectable face.	
ID32_fusion_ ID3_foreground_	Maximum number of FAST keypoints based on all frames of the video for the image foreground.	The assumption for this set of features is that an almost constant value can be found throughout the course of the video. As a result, no significant differences between minimum and maximum of each feature are expected. Greater distances are seen as an indication of DeepFakes. Normalization is carried out on the basis of the two representations *Face* and *ROI* (see Figure 6 for reference) based on the level of detail as well as the number of pixels. Formally, this takes the form of $\frac{FeatureFace}{FeatureROI}$, where $FeatureFace|ROI=\frac{FeatureCountFace|ROI}{PixelcountFace|ROI}$. In order to prevent division by 0, the default value is set to −1 to be outside the considered range.
ID33_fusion_ ID4_foreground_	Minimum number of FAST keypoints based on all frames of the video for the image foreground.	
ID34_fusion_ ID5_foreground_	Maximum number of SIFT keypoints based on all frames of the video for the image foreground.	
ID35_fusion_ ID6_foreground_	Minimum number of SIFT keypoints based on all frames of the video for the image foreground.	
ID36_fusion_ ID7_foreground_	Maximum number of Sobel edge pixel based on all frames of the video for the image foreground.	
ID37_fusion_ ID8_foreground_	Minimum number of Sobel edge pixel based on all frames of the video for the image foreground.	
